# Fracture of Patella

**Published:** 1888-09

**Authors:** W. G. Jameson

**Affiliations:** Rusk, Texas


### FRACTURE OF PATELLA.

By W. G. Jameson, M. D., Rusk, Texas.

[Read in Section on Surgery and Anatomy T. S. M. A., at Galveston. Received too late to appear in Transactions, and published in the JOURNAL by authority of the writer.]

ACCORDING to statistics furnished by a number of surgeons this bone comprises less than two per cent. of all fractures treated. It is a small flat sesamoid bone, resembling the common figure of a heart, with its point downward. It is placed in front of the knee, to give protection to joint and also leverage to the quadratus femoris muscle in performing the various movements of the leg. At the upper border we have the conjoined tendon of the great quadratus femoris muscle; attached below is the ligamentum patellæ, which is a continuation of the conjoined tendon, and on either side the capsular ligament. With these tendonous attachments the patella is kept in its proper place and made to perform its duties. It also has a prepatella fibrous envelope surrounding it, which plays an important part in this fracture, according to Dr. MacEwen, of Glasgow, who, I believe, was the first to call attention to this new feature in its pathology. The point in question, as demonstrated, is, that when solution of the patella takes place there is also more or less laceration of the pre-patellal fibrous and aponeurotic structures, which fall between the fragments, preventing bony union. When the laceration is but slight, the chances for osseous union is much better. I know of no fracture that gives the surgeon more cause for anxiety as to its ultimate results and future usefulness of limb. Our object in treating fractures is to restore the parts to as near normal as possible. The majority of the works on surgery say that we seldom ever get bony union. Dupuytran, who thought he had obtained bony union, offered for patella of his patients its weight in gold; the union, in the majority of cases, being ligamentous. Numerous appliances have been devised by surgeons for treating this fracture. Recently some have advised converting the simple fracture into a compound one, by cutting down on fractured ends and wiring them together in order to get bony union, the operation being performed under strict antiseptic precaution, while others condemn the method as being dangerous, not only to limb, but life of the patient. Dr. Wyeth, in his admirable work on surgery, gives his experience with the operation; only saved the life of his patient by sacrificing the limb. He condems it as being hazardous. It was not my intention to enter into a discussion as to the various modes of treating this fracture, and their relative merits.

The case I am about to report is that of a robust young man, E. T. D., age thirty, while trying to board a freight train, moving at the rate of ten miles per hour, fell, striking the right knee against the end of the cross tie, fracturing the patella transversely, near junction of middle with lower third. Also fractured piece as large as chestnut from inner edge of bone vertically connecting with the transverse. He was carried to Lufkin when first injured, where he received medical attention byDr. Denman. Next morning he was brought to Rusk. I first saw him with Dr. J. K. Sturdevant, about twelve hours after receiving injury. He was then complaining of pain in parts. The temporary dressing being removed, we found the joint very much swollen, and upon manipulation easily detected the fracture. The fragments were separated but little, showing that the pre-patella fibrous tissue and capsular ligament had sustained but slight injury. There was considerable effusion into joint, which no doubt gave rise to separation and is one hindrance to bony union. Just over the patella the soft parts were ecchymosed. The dressing used was Hamilton's, the material for splint sole leather, which extended from the gluteal fold to near the heel, and enveloped the posterior half of limb. The splint was well soaked in warm water, so as to mold it perfectly to the parts. The splint was enclosed in a cloth bandage and well padded with cotton batting. The bandages used were made of soft cotton cloth, the roller was applied to the lower end of the splint and continued to within three inches of the knee joint, and the upper end of the splint was bandaged to the limb to within the same distance of the knee; the next roller was fixed above the patella by several circular turns, and then carried below, where it was carried around the limb again. It was then passed alternately above and below, until the whole knee was covered; at the same time the fragments of bone were firmly pressed together by Dr. Sturdevant. The bandage was applied just comfortably tied, the end being secured by a safety pin. The roller was stitched the whole length of the splint on each side to the cloth sack enveloping the splint. He was put to bed with the foot elevated about eight inches, so as to relax the quadriceps muscle as much as possible. Bowels opened with Seidlitz powders. After dressing the parts he felt more comfortable, complained of no pain, slept well. The fifth day the bandage around the knee began to get loose; it was partially removed, the parts inspected and reapplied, just tight enough to feel comfortable and hold the parts together. We continued to readjust until the effusion was entirely removed. He was confined to his bed two weeks and to the room four. After that time we removed the entire dressing and began passive motion. The fragments were accurately adjusted and seemed to be united by bone—the joint motion is very limited, almost completely anchylosed. While I gently flexed the limb, Dr. Sturdevant supported the fractured parts so as to prevent reseparasion. The patella seemed firmly fixed, but by manipulation the adhesions gave way and soon became moveable. The passive motion was repeated three times per week—the joint motion improving each time. After the sixth week he was allowed to go on crutches. The long splint was worn about two months. It was then cut off to eighteen inches in length and used about two weeks. He left it off against our advice and began walking with a cane. He gained the use of the limb rapidly and was soon able to flex it as well as the other. The union, so far as we are able to judge, is bony. We cannot, by making direct pressure, detect any separation. In no way you apply pressure to the patella can you get motion of bone only en masse. We cautioned him, in case it might be ligamentous, that by use there might, and doubtless would be separation of fragments. It has now been over three months since he began using a cane without any support to the knee. He can walk without it very well. There is no separation and the parts feel firmly united. The patella is slightly enlarged, but as freely moveable as the other. When the bandage was first removed the muscles were atrophied, but each time passive motion was used, we had the parts well kneaded. There is just a perceptible limp in the walk, which is no doubt due to habit. I see no difference in flexing. The limb is now as plump as the other. He can go up or down steps slowly without putting both feet on the same step, which cannot be accomplished when a union is ligamentous. The functions of a quadriceps muscle are all well executed, especially when an attempt is made to perform such movements as are solely executed by that muscle, such as bringing the foot forward with knee held rigid, which cannot be done except with bony union—the other, when lying on bed to lift the limb without bending the knee.

